# CORRECTION

**DOI:** 10.1111/1759-7714.14802

**Published:** 2023-01-23

**Correction to genetic variants in LKB1/AMPK/mTOR pathway are associated with clinical outcomes of chemotherapy in non‐small cell lung cancer**

In Sun Ha Choi et al.^1^ the following error was published on page 3322.

The authors noticed that the funding statement is missing. The Funding information section should read:

## Funding information

This research was supported by Kyungpook National University Research Fund, 2021.

The publisher apologizes for the error and any inconvenience it may have caused.
